# Pemphigus Vulgaris: A Clinical Study of 31 Cases (2004–2014) in Morocco

**DOI:** 10.1155/2020/8535109

**Published:** 2020-09-08

**Authors:** Titou Hicham, Fatima Zahra Chahnoun, Tarik Hanafi, Naoufal Hjira, Boui Mohammed

**Affiliations:** Dermatology Department, Military Hospital Instruction Mohammed V, University Mohammed V, Rabat, Morocco

## Abstract

**Background:**

Pemphigus vulgaris is a rare bullous autoimmune dermatosis whose evolution and prognosis are unpredictable.

**Aim:**

The objective was to analyze long-term outcomes in patients with pemphigus vulgaris by identifying the factors that are able to influence prognosis, in particular the phenotype of pemphigus vulgaris, age at onset, multiplicity of mucosal involvement, relapse and remission rates, and survival functions.

**Methods:**

A retrospective analysis of a cohort of 31 patients followed for pemphigus vulgaris during the period from January 2004 to January 2014. Inclusion criteria were a diagnosis of pemphigus vulgaris confirmed by histopathology and direct immunofluorescence (DIF) and a period of follow-up of at least five years from the diagnosis. The following information was collected by a single investigator.

**Results:**

In total, 67.7% of patients presented a mucocutaneous pemphigus vulgaris. Male-female sex ratio was 2.4. The median duration of patient's follow-up was estimated at 7 (6–9) years. Multiple mucosal involvement in the oral cavity and at other mucosal sites was significantly associated with severe mucocutaneous pemphigus vulgaris (*p*=0.01). Multiple relapses were significantly associated with the disease severity (*p*=0.04).

**Conclusion:**

Poor prognosis factors were severe mucocutaneous type of pemphigus vulgaris and multiple mucosal involvement in the oral cavity and at other mucosal sites.

## 1. Introduction

Pemphigus vulgaris (PV) is a rare chronic bullous disease of the skin and mucous membranes. It is clinically characterized by blisters and erosions of the mucous membranes and skin.

PV is an organ-specific autoimmune disease characterized by the production of autoantibodies directed against desmosomal proteins leading to acantholysis and thus the forming of epidermal bullae [1, 2].

Pemphigus vulgaris is the most frequent form of pemphigus with more than 70% of cases [3]. PV shows unequal geographical and ethnic distribution with annual incidence rates ranging from 0.76 to 16.1 per million of inhabitants [4, 5]. The classical treatments of pemphigus are corticosteroids associated or not with immunosuppressive agents [6, 7]. The treatment objective consists in inhibiting the occurrence of new lesions with minimal or nonexistent treatment. The management of PV is difficult because of frequent recurrence and the occurrence of complications linked to the disease evolution, treatments, and probably to other factors linked to the patient such as comorbidity [8, 9].

The disease prognosis was revolutionized by the introduction in the 1950s of corticosteroids, which decreased mortality from 75 to 80 to 30%. Currently, the disease remains associated with a mortality rate of about 6% despite the use of various adjuvant treatments [10, 11]. This mortality is essentially attributed to the side effects of used treatments, corticosteroids, and immunosuppressants [12]. A therapeutic challenge of this last decade was the research of new treatments able to replace corticosteroids and presenting less important side effects [13, 14]. The objective of this study was to analyze the long-term outcomes of patients with PV by identifying the factors able to influence the prognosis, in particular the phenotype of PV, age at onset, multiplicity mucosal involvement, recurrence and remission rates, and survival functions.

## 2. Materials and Methods

An analytical monocentric study led on a retrospective cohort of patients with PV and followed up at dermatology department of Rabat Military Hospital from January 1^st^, 2004 to January 1^st^, 2014. In this analysis, inclusion criteria were a diagnosis of pemphigus vulgaris confirmed by histopathology and direct immunofluorescence (DIF) and a period of follow-up of at least five years from the diagnosis. Indirect immunofluorescence (IIF) studies were repeated every six months or in case of clinical relapse. On the other hand, direct immunofluorescence was repeated after at least 12 months of absence of symptoms in patients with PV. The following information were collected by a single investigator: medical history, similar familial diseases, sociodemographic characteristics (age and sex), clinical data (age at onset of disease, phenotype of PV, received treatment, duration of remission, and number of relapses), and date in the latest news. Clinical status at the onset was evaluated retrospectively, thanks to the medical records and the patients' photographic documentation. The Autoimmune Bullous Skin Disorder Intensity Score (ABSIS) was used to evaluate the disease severity [15]. In the ABSIS, the results were summed to obtain a total score ranging from 0 to 206, which takes into account the cutaneous involvement (0–150), oral involvement (0–11), and subjective discomfort during eating and drinking (0–45). The total scores classified PV cases into three levels of severity: mild PV (scores from 1 to 10); moderate PV (scores from 11 to 40); and severe PV (scores from 41 to 206).

### 2.1. Statistical Analysis

Statistical analysis was made using the Statistical Package for Social Sciences (SPSS) Package version 20.0. Student's test was used to compare the continuous variables, and the chi-squared test was used to compare the proportions between independent groups. Asymmetric distribution quantitative variables were analyzed by the Mann–Whitney test to compare two independent groups, and the Kruskal–Wallis test was used to compare more than two independent groups. The Kaplan–Meier method was used to estimate survival probability according to duration of remission. To compare survival curves according to sex, the log-rank test was used. The different variables were calculated with a confidence interval (CI) of 95%. *p* value lower than 0. 05 was considered as significant.

## 3. Result

### 3.1. Epidemiological Data

In total, 31 patients were included in this study with male dominance (70%). The average age at onset of disease was 54.1 ± 14.1 years (extremes: 33–80 years) in male patients and 55.1 ± 14.4 years (extremes: 35–73 years) in female patients. The difference in the average age between the two sexes was not statistically significant (*p*=0.48). The average duration of the follow-up was 7.2 ± 1.79 years (extremes: 5–10 years). Patients were seen between two weeks and 10 months (median: 4 (3–6) months) after the onset of symptoms, and about the quarter (22.5%) of patients were seen during the first six months. None of the patients had a family history of PV.  Table 1 represents the patients' epidemiological and clinical characteristics (sex, age at onset, phenotype of PV, and severity of disease).

Oral mucous lesions were observed in 22 patients (70.9%) independently from the severity of disease (*p*=0.56). About half of the patients presented severe PV (51.6%), but no one of those with mild PV were afflicted in multiples mucosal sites (*p*=0.01).

A high ABSIS score (22.5) of oral involvement was only documented in patients with severe PV (27.3%). The involvement of other mucous membranes was observed in 9 (29%) patients including membranes in nasal (*n* = 3 patients; 9.6%), genital (*n* = 3 patients; 9.6%), laryngeal (*n* = 3 patients; 9.6%), rectal (*n* = 2 patients; 6.4%), conjunctival (*n* = 2 patients; 6.4%), and esophageal (*n* = 1 patient; 3.2%). However, other mucosal involvement was more frequent in patients with severe PV of the mucocutaneous type than in patients with moderate or mild PV (*p*=0.01).

Oral cavity involvement was more severe in female patients than in male patients (55.3% vs. 44.2%) as well as other mucous membranes involvement (29.5% vs. 40.5%). In our series, the initial lesion site was oral cavity in 22 (70.1%) patients. Cutaneous initial lesion was more frequently documented at the level of members and head (*n* = 4 patients; 12.9%).

The average age of patients with mucous initial lesion was 52 ± 13.7 years and that of patients with cutaneous initial lesion was 60.4 ± 14.3 years. The difference was not statistically significant (*p*=0.15).

### 3.2. Treatment and Evolution

In this study, all the patients were initially treated by systemic corticosteroid therapy at a dose of 1–1.5 mg/kg (*n* = 23; 74%), 1–2 mg/kg (*n* = 6; 19.4%), or >2 mg/kg (*n* = 2; 5.6%). This last dose was administered in intravenous bolus. Corticosteroid therapy was administered in monotherapy in 26 (83.8%) patients and in association with immunosuppressive adjuvant treatment in 5 (16.1%) patients. The adjuvant treatment was associated to strengthen the immunosuppressive action and reduce the side effects of systemic corticosteroids in patients insufficiently responded to the treatment. Adjuvant treatment was used more frequently in the case with severe PV than in the case with moderate or mild PV (*p*=0.01). Azathioprine was administered in four (12.9%) patients. However, the remaining patients were treated by methotrexate (3.2%). Rituximab was used as a second-line therapy in only one patient who did not respond to systemic corticosteroid therapy associated with azathioprine. A remission was obtained after two cures of 1 g of rituximab in a two-week interval. Remission was obtained after an average duration of therapy of 4.1 ± 1.7 months: 1 month in six (19.4%) patients, 1-2 months in ten (32.3%) patients, and >2 months in fifteen (48.4%) patients. The duration of the first remission was <2 years in two (6.4%) patients.

### 3.3. Relapses and Survival

**Figure 1:**
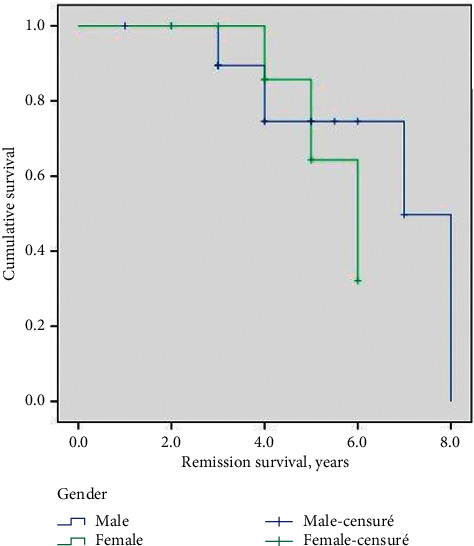
Survival in remission in male and female patients using Kaplan–Meier. Data are censored for patient deaths during the study period.

Disease relapse was defined as the appearance of at least three new lesions per month, which did not regress spontaneously over a week or as an already existing lesion in a patient who achieved the disease control. One or two relapses were documented in 8 (25.8%) patients, and three or more relapses were documented in one (3.2%) patient during the first five years of follow-up. The number of relapses was statistically correlated with PV severity score. Multiple relapses were more frequently associated with severe PV (9/16; 56.2%) than with moderate PV (2/12; 16.6%). No patient with mild PV presented multiple relapses (*p*=0.04). In a patient (3.2%), relapse was documented after 8 years of complete remission without therapy. The cause of his death was a cardiorespiratory arrest following severe sepsis. The average duration of survival in remission in women was longer than in men (4.3 years vs. 4.1 years; *p*=0.3). This difference was not statistically significant (Figure 1).

Table 2 summarizes the differences in the survival duration in remission by comparing several subgroups according to sex, age, severity of PV, phenotype of PV, and mucous involvement site. Disease activity decreased with time in most patients (Table 3). After 5 years of follow-up, 7 (22.6%) patients were in complete remission without treatment. Two (6.5%) patients were in complete remission after two years. A total of 21 (67.7%) patients received a minimal dose, and three (9.6%) patients received high-dose therapy. Immunological remission (established by IIF) followed clinical remission within 6–12 months. A DIF examination was demanded after a year of complete remission and negative IIF examination. A negative DIF result indicated the immunosuppressive treatment stopping.

### 3.4. Treatment Complications and Death

During the period of follow-up, corticosteroids side effects were observed in 26 (83.9%) patients. The most common side effects were osteopenia and osteoporoses (*n* = 15; 48.4%), diabetes (*n* = 9; 29%), cataract (*n* = 8; 25.8%), candidiasis (13; 41.9%), hypertension (*n* = 16; 36.4%), weight gain (*n* = 7; 22.5%), Cushing's syndrome (*n* = 8; 25.8%), and mood change (*n* = 9; 29%). Side effects of adjuvant treatment were observed in two patients (6.4%) who used azathioprine (leucopenia) or methotrexate (hepatopathy). Three patients (9.7%) died following a cardiovascular accident, 2 (6.4%) patients following ischemic heart disease, and 1 (3.2%) patient following severe sepsis. The causes of death were not directly assigned to PV in any patient.

### 3.5. Clinical Status at the End of Follow-Up

After five years of follow-up, 7 (22.5%) patients were not under treatment. These patients were in complete remission after treatment for an average duration of 6.1 years (extreme: 2–8 years). 21 (67.7%) patients were in remission under minimal therapy (Table 3). In the 26 patients who remained alive at the end of the study, prednisone was used as maintenance treatment at a dose of 5 mg (*n* = 16; 51.6%) or at a dose of 10 mg (*n* = 8; 25.8%).

At the end of the study, clinical status in 26 patients who survived was reflected by an ABSIS score of 0 in 18 (69.2%) patients. Indirect immunofluorescence was negative in 16 (61.5%) patients in the end of the study.

## 4. Discussion

Our work reports a 10 years' follow-up of a cohort of 31 patients with pemphigus vulgaris in Morocco. Our method of data collection was done from three sources (medical record, contact by phone, and administrative files) to ensure exhaustiveness. The objective of this study was to identify the parameters allowing to forecast the results and prognosis in patients followed for PV. Therefore, we analyzed the parameters such as age at onset, sex, initial severity, relapse rates, and survival in remission.

The results of the present study were comparable with those of previous studies. In our series, PV incidence peak was observed in the fifth decade of life. Previous studies reported a similar distribution in the fourth and fifth decades [16–18]. More specifically, a higher prevalence of PV was noticed in Mediterranean origin and Jewish patients [19].

In the present study, the average age was 54 ± 14.2 years. In relation to our results, studies found a similar average age [17, 18, 20], and other studies carried out in the Middle East and Arabic countries mentioned a higher average age, particularly in Iran (42 years) [21], Turkey (43 years) [22], Kuwait [23], and Saudi Arabia (43.1 years) [24]. In our study, the average age at onset of PV was comparable between two sexes (55.1 years vs. 54.1 years). This difference in the average age was not statistically significant. Indeed, major previous studies found similar results [16, 25, 26].

In the present study, the proportion of male patients (70%; sex ratio 2.4) was more important. A similar result was reported in Spain with a sex ratio of 1.22 [26]. However, several studies mentioned female dominance [16, 17, 25]. The professional profile of our cohort could explain in part male dominance. In our study, about two-thirds of patients (67.7%; 21/31) presented a mucocutaneous type of PV. A similar result was reported in Bulgaria (64.8%) [27] and in Iran (70.8%) [28]. The initial involvement of oral mucosa was present in major patients (70.9%; 22/31). A similar result was reported in previous cohorts [16, 27]. In our study, the severity of oral involvement was statistically associated with the severity of PV (*p*=0.01). Some authors considered the initial involvement of oral mucosa as a marker of poor prognosis [29, 30]. Indeed, the severity of oral mucous involvement is correlated with anti-Dsg3 antibodies rate [31]. The involvement of other mucous membranes (larynx, esophagus, conjunctiva, and genital) in the patients with PV was only rarely reported in literature. Therefore, we estimate that their repercussions were probably underestimated. In our series, involvement of other mucous was more frequent in patients with severe mucocutaneous type of PV than in patients with moderate or mild PV (*p*=0.01). The involvement of genital mucosa was reported as second in terms of frequency in certain previous cohorts [16, 32]. In our study, laryngeal involvement was present in 3 patients (9.9%). A similar result was reported in Italy (10.2%) [18] and Slovakia (13.6%) [16]. However, certain authors mentioned laryngeal involvement in 55.3% of patients [33].

Strategies for the management of PV aim to achieve and maintain a complete remission while minimizing side effects. In the absence of established international recommendations, the initial dosage of corticosteroids and the choice of adjuvant choices reflect the clinicians experience at different medical centers. In our center, we adapted the initial dose of systemic corticosteroids to severity of PV. A long-term oral corticosteroid therapy was often necessary to control PV of our patients. Many studies reported a similar practice regarding the adaptation of initial dose of corticosteroids and the choice of the adjuvant treatment according to disease severity [16, 34].

The use of adjuvant treatment was more frequent in severe PV than moderate or mild PV. Rituximab was exclusively used in patients with refractory PV. To the present day, rituximab is known as a sure and efficient treatment for the disease refractory forms [35]. The side effects of the medications used to treat PV were frequent in previous cohorts [16, 34]. In the present study, side effects were observed in 26 (83.9%) patients. Arterial hypertension (36.4%) and diabetes mellitus (29%) were the most observed complications in our patients. Similar results were reported in previous studies [11, 34]. Our results found a statistically significant association between the number of relapses and severity of PV (*p*=0.04). Indeed, multiple relapses were more frequent in patients with severe PV than in patients with moderate PV (16.6% vs. 56.2%; *p*=0.04).

However, no patient with mild PV presented multiple relapses. In our study, factors triggering relapses were dominated by infections (12.9%) followed by corticosteroids regression (9.7%) and stress (6.4%). However, the triggering factor was not identified in 19.3% of our patients. Similar results were reported in previous cohorts [11, 16, 34].

The mode of arrest of corticosteroids and immunosuppressive medications remain an active area of debate. In the present study, major patients obtained complete and long-lasting remission by a standard therapy. Indeed, 7 (22.6%) patients were in complete remission after 5 years, and 2 patients (6.4%) were in complete remission after 2 years. However, 2 patients (6.4%) did not obtain complete remission by remaining in partial remission under high-dose treatment after 5 years. The study carried out in Slovakia [16] found similar result: 2.3% of patients were in remission after two years, and 18.2% after 5 years. The highest remission rate was reported by the study carried out in New York [36]: 25% and 50% of patients were in remission after 2 and 5 years, respectively.

Size difference of severe PV subgroups and multiplicity of the disease evaluation method could explain in part the fluctuations in remission rate in previous studies. All the patients included in our study were subject to continuous follow-up for an average of 7.2 years. Our results promise to consider PV as a good prognosis disease, since more than 50% of patients were in complete remission after a follow-up of 2 or 5 years. Median remission survival in female patients was slightly more longer than male patients (4.3 years vs. 4.5 years, *p*=0.3). The difference was not statistically significant. A similar result was reported in previous studies [29].

Certain factors of poor prognosis were reported in previous studies such as young age at the onset of disease (<40 years), female sex, and initial mucous involvement [6, 29, 30].

Our study limits were the retrospective character and the small size of the sample. Indeed, the rarity of disease and the monocentric character of this study limited the sample size. ABSIS score was chosen by our team to evaluate the disease severity since it allows a precise definition of PV different phenotypes and facilitates the comparison with the results of other studies. This work is the first of its kind in Morocco. Its other merit is having evaluated this disease over a period of ten years.

## 5. Conclusion

The present study reveals certain factors which seem to play a role in determining the poor prognosis, such as the severe mucocutaneous type of pemphigus vulgaris and multiple mucosal involvements in the oral cavity and other mucosal sites.

## Figures and Tables

**Table 1:** Characteristics of patients: age at onset, sex, and phenotype of pemphigus vulgaris (PV).

Patients	Age at onset, years, average ± SD	Phenotype, *n* (%)	M : F ratio
All (*n* = 31)	54 ± 14.2	Mucocutaneous 21 (67.7%)	2.4
		Mucosal 7 (%)	
		Cutaneous 3 (9.7%)	

Mild (*n* = 3)	57 ± 16.4	Mucocutaneous 1 (33.3%)	2
		Mucosal 2 (66.7%)	
		Cutaneous 0 (%)	

Moderate (*n* = 12)	47.7 ± 20.8	Mucocutaneous 8 (66.7%)	3
		Mucosal 2 (16.7%)	
		Cutaneous 2 (16.7%)	

Severe (*n* = 16)	54.9 ± 14.1	Mucocutaneous 12 (75%)	2.2
		Mucosal 3 (18.8%)	
		Cutaneous 1 (6.3%)	

SD, standard deviation; M, male; F, female.

**Table 2:** Remission survival in patients with pemphigus vulgaris (PV).

	Patients, n	Remission survival, years, median (95% CI)	*p*
Age, years			
≤40	8	3.5 (3–5)	0.42
>40	23	4 (3–5)	
≤50	14	3 (3–5)	0.06
>50	17	4 (3–6)	

Gender			
Male	22	4 (3–5)	0.28
Female	9	4 (3–5)	

Severity of PV			
Mild	3	3 (2–5)	0.67
Moderate	12	4 (3–6)	
Severe	16	4 (3–5)	

Phenotype of PV			
Mucocutaneous	24	4 (3–5)	0.30
mucosal	7	3 (3–6)	

Primary mucosal involvement			
Present	22	4 (3–5.12)	0.07
Absent	9	3 (2–5)	

Other sites of mucosal involvement			
Present	13	4 (3–5.5)	0.36
Absent	18	4 (3–5.12)	

95% CI, 95 % confidence interval.

**Table 3:** Remission rates in patients with pemphigus vulgaris (PV) and indirect immunofluorescence (IIF) results obtained after 2 and 5 years of follow-up.

Patients	Remission	2 years	IIF-negative	5 years	IIF-negative
All	CRNT	2 (6.4)	2 (6.4)	7 (22.6)	7 (22.6)
	CRMT	17 (54.8)	6 (19.3)	14 (45.2)	6 (19.3)
	PRMT	10 (32.2)	0	7 (22.6)	0
	PRHT	2 (6.4)	0	2 (6.4)	0

Mild PV (*n* = 3)	CRNT	1 (33.3)	1 (33.3)	2 (66.6)	2 (66.6)
	CRMT	2 (66.7)	2 (66.6)	0	0
	PRMT	0	0	0	0
	PRHT	0	0	0	0

Moderate PV (*n* = 12)	CRNT	1 (8.3)	1 (8.3)	3 (25)	3 (25)
	CRMT	8 (66.6)	2 (16.6)	8 (66.6)	5 (41.6)
	PRMT	3 (25)	0	1 (8.3)	0
	PRHT	0	0	0	0

Severe PV (*n* = 16)	CRNT	0	0	1 (6.25)	1 (6.25)
	CRMT	8 (50)	1 (6.25)	4 (25)	0
	PRMT	6 (37.5)	0	8 (50)	0
	PRHT	2 (12.5)	0	2 (12.5)	0

CRNT, complete remission without (no) therapy; CRMT, complete remission under minimal therapy; PRMT, partial remission under minimal therapy; PRHT, partial remission under high-dose therapy.

## Data Availability

The data used to support this study are available within the article and are made available from the corresponding author upon request.
